# Granulomatous/sarcoid-like lesions associated with checkpoint inhibitors: a marker of therapy response in a subset of melanoma patients

**DOI:** 10.1186/s40425-018-0323-0

**Published:** 2018-02-12

**Authors:** Michael T. Tetzlaff, Kelly C. Nelson, Adi Diab, Gregg A. Staerkel, Priyadharsini Nagarajan, Carlos A. Torres-Cabala, Beth A. Chasen, Jennifer A. Wargo, Victor G. Prieto, Rodabe N. Amaria, Jonathan L. Curry

**Affiliations:** 1 Department of Pathology, Section of Dermatopathology, The University of Texas MD Anderson Cancer Center, Houston, TX USA; 2 Department of Translational and Molecular Pathology, The University of Texas MD Anderson Cancer Center, Houston, TX USA; 3 Department of Dermatology, The University of Texas MD Anderson Cancer Center, Houston, TX USA; 4 Department of Melanoma Medical Oncology, The University of Texas MD Anderson Cancer Center, Houston, TX USA; 5 Department of Pathology, Section of Cytopathology, The University of Texas MD Anderson Cancer Center, Houston, TX USA; 6 Department of Nuclear Medicine, The University of Texas MD Anderson Cancer Center, Houston, TX USA; 7 Department of Surgical Oncology, The University of Texas MD Anderson Cancer Center, Houston, TX USA

## Abstract

**Background:**

Immune checkpoint therapy has dramatically changed the landscape of cancer therapy, providing an efficacious and durable therapeutic option for patients with advanced-stage disease. However, dermatologic toxicities are a well-recognized side effect in patients receiving this therapy. A spectrum of immune related adverse events (irAEs) involving the skin can occur and include immunobullous disorders, lichenoid dermatitis, and vitiligo. Granulomatous/sarcoid-like lesions are now being recognized with the current class of checkpoint inhibitors (CPIs) that involve the dermis, the subcutaneous tissue (panniculitis), and lymph nodes.

**Case presentation:**

We report 3 patients who developed granulomatous/sarcoid-like lesions while being treated with immune checkpoint therapy for advanced-stage melanoma, and we provide a comprehensive review of the literature in which similar cases are described. To date, 26 patients (including the 3 from this report) have been described with a median age of 57 years who developed granulomatous/sarcoid-like lesions associated with CPIs (median onset 6 months), of which 77% of patients had melanoma as primary tumor. To manage this adverse side effect, therapy was withheld in 38% of patients and 44% of the patients were treated with systemic steroids and 8% patients with localized therapy (one patient with intralesional triamcinolone). 96% of patients demonstrated either resolution or improvement of granulomatous/sarcoid-like lesions associated with CPIs irrespective of medical intervention. Therapeutic response, stable disease, or remission of primary malignancy was observed in 71% of reported patients who developed granulomatous/sarcoid-like lesions associated with CPIs over a median follow-up of 11.5 months since initiation of treatment.

**Conclusions:**

The development of granulomatous/sarcoid-like lesions associated with CPIs is a recognized manifestation with the current class of immune checkpoint therapy that may clinically and radiographically mimic disease recurrence. Awareness of this type of toxicity is important for appropriate management and possible measurement of therapeutic response in a subset of patients who manifest this type of immune-mediated reaction.

## Background

Immune checkpoint inhibitors (such as ipilimumab, nivolumab and pembrolizumab) are novel monoclonal antibodies that target cytotoxic T-lymphocyte–associated protein 4 (CTLA-4) and programmed cell death protein 1 (PD-1) cell signaling, respectively and attempt to restore patients’ anti-tumor T-cell response that may be endogenously diminished as part of tumor escape mechanisms. Inclusion of immune checkpoint inhibitors in the armamentarium to battle cancer has broadened the landscape of cancer therapy since their use can elicit clinically efficacious and durable anti-tumor immune response [1, 2].

Cancer patients receiving immune checkpoint inhibitors are prone to develop immune-related adverse events (irAEs); a common and early site of involvement includes the skin [3]. The types of dermatologic toxicities are diverse and include dermal hypersensitivity reactions, lichenoid eruptions, and immunobullous reactions and may become sufficiently severe to require cessation of further treatment [4–6]. Granulomatous/sarcoid-like lesions are a recognized toxicity associated with the current class of therapy with checkpoint inhibitors (CPIs) that can involve the dermis and subcutis (erythema nodosum-like panniculitis) [7–11]. Granulomatous/sarcoid-like lesions associated with CPIs are significant because they often mimic disease recurrence and/or consequently lead to cessation of therapy, adding further challenges to the management of adverse immune-related events.

Systemic sarcoidosis is a multi-organ disease of unknown etiology in which there is collection of epithelioid immune cells in the form of granulomata in affected tissue [12]. Sarcoidal granulomata are frequently located in the lungs, lymph nodes, cardiovascular sites, central nervous system, and the skin [13]. Pulmonary granulomata and hilar lymphadenopathy (LAD) may be seen in 90% of patients with sarcoidosis [13]. A diagnosis of sarcoidosis requires demonstration of the characteristic granulomata on tissue biopsy, and/or by a constellation of clinical symptoms, including fever, fatigue, shortness of breath and weight loss, which may indicate specific organ involvement [12]. Granulomatous/sarcoid-like lesions associated with CPIs may exhibit histopathologic features of “sarocoidal” granulomas and may represent reactions to therapy [14].

We report 3 patients who manifested clinical and radiographic features of granulomatous/sarcoid-like lesions associated CPIs. Awareness of this toxicity from the current class of CPI will be critical for appropriate diagnosis and patient management.

## Case presentation

### Patient 1

A 79-year-old man with stage IV M1C *NRAS Q61K*-mutant metastatic melanoma from an ulcerated primary lesion on the chest (Breslow thickness, not available due to tangential sections, Clark level IV, 26 mitotic figures/mm^2^), and metastasis to 1 of 40 regional lymph nodes without extracapsular extension, demonstrated an enlarging right lung nodule 8 months after his initial diagnosis. Given concern for disease progression, he completed 4 doses of ipilimumab without evidence of therapeutic response. He had no history of autoimmune disease. Past medical history included type 2 diabetes mellitus, hypertension, and mixed hyperlipidemia.

**Fig. 1:**
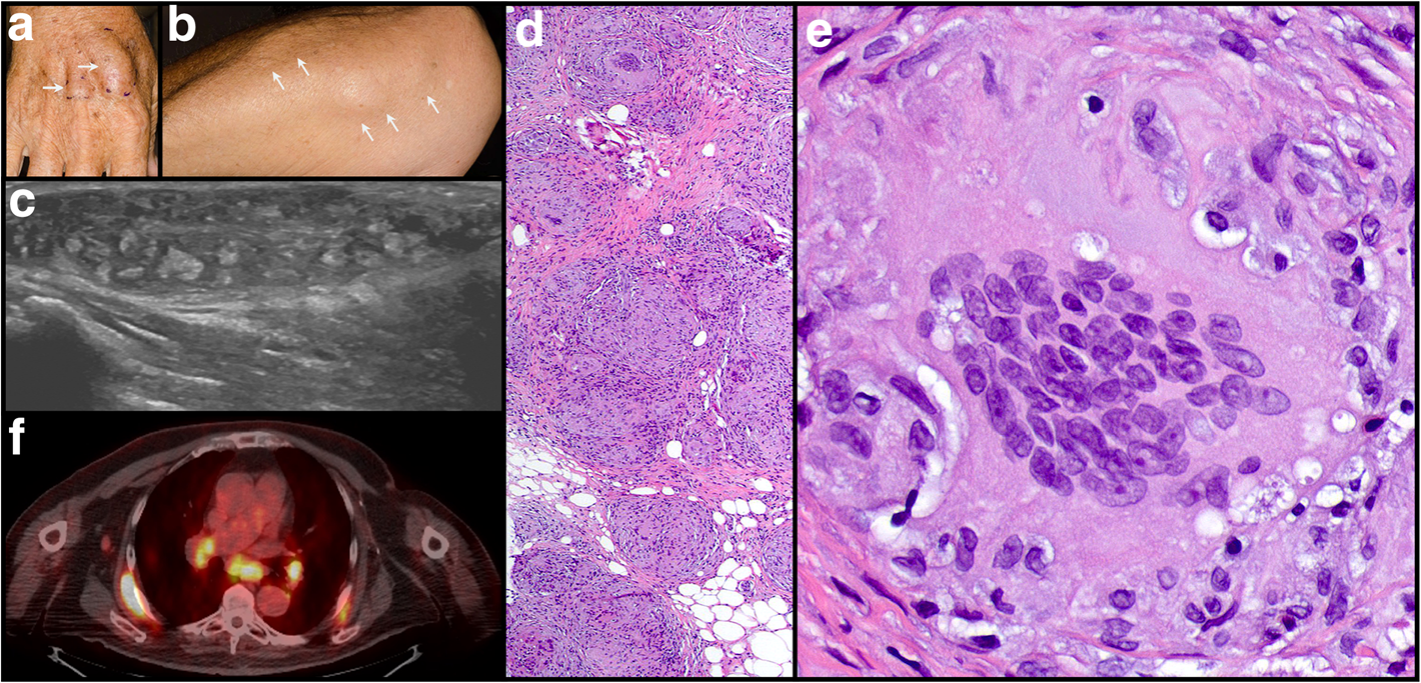
Patient 1. Multiple soft tissue nodules on the **a** wrist (arrows) and **b** forearm (arrows). **c** Ultrasound of wrist with a nodular plaque. **d** Biopsy of nodule with non-caseating granulomata in the dermis and subcutis composed of a collection of epithelioid histiocytes (hematoxylin and eosin [H&E] original magnification × 40). **e** Granulomata with multinucleated giant cells (H&E original magnification × 400). **f** PET/CET with FDG avid bilateral hilar and mediastinal lymph nodes

Six months after completion of ipilimumab therapy, surveillance studies revealed interval progression of disease with new bilateral pulmonary nodules, mediastinal and hilar lymphadenopathy, a liver lesion, and an adrenal nodule, suggestive of metastatic melanoma. Pembrolizumab therapy (2 mg/kg) was subsequently initiated, and computed tomography (CT) scans 3 months after initiation of pembrolizumab revealed radiographic improvement and decreased size of pulmonary, liver, and adrenal lesions. The patient tolerated 22 cycles of pembrolizumab with minimal adverse reactions, and CT scans obtained 15 months after initiation of therapy revealed stable disease without new lesions. Restaging positron emission tomography/CT (PET/CT) scans obtained 18 months after initiation of pembrolizumab therapy revealed enlarging gastric, retroperitoneal, and paratracheal lymph nodes, and after 20 months of pembrolizumab therapy (27 cycles), the patient presented with erythema and swelling of the forearms. Physical examination revealed symmetrical, firm subcutaneous nodules involving bilateral dorsal hands, forearms, and elbows (Fig. 1a-b). Ultrasound examination revealed a 4.3 × 1 × 2.3 cm soft tissue nodule (Fig. 1c), and needle core and skin punch biopsies showed a collection of epithelioid histiocytes forming non-caseating granulomata (Fig. 1d-e). Fite, Gomori methenamine silver, and gram stains and tissue cultures were negative for microorganisms. Melanoma was not identified. PET/CT performed at 23 months of pembrolizumab therapy (30 cycles), revealed prominent fluorodeoxyglucose (FDG) avidity of mediastinal / bilateral hilar LAD and subcutaneous nodules (Fig. 1f). In view of the clinical, radiographic, and histologic findings, these features were consistent with a cutaneous granulomatous/sarcoid-like lesions associated with CPI involving the skin and pulmonary lymph nodes. Given the patient’s excellent response to pembrolizumab therapy and the development of these therapy related granulomatous/sarcoid-like lesions, his therapy was subsequently discontinued.

Follow up examination at 2 months demonstrated persistence of skin nodules and intralesional triamcinolone was initiated. Restaging studies at this time demonstrated stable LAD and no evidence of melanoma disease progression. One month later, intralesional injection demonstrated interval softening and decreased diameter of the cutaneous nodules.

### Patient 2

**Fig. 2:**
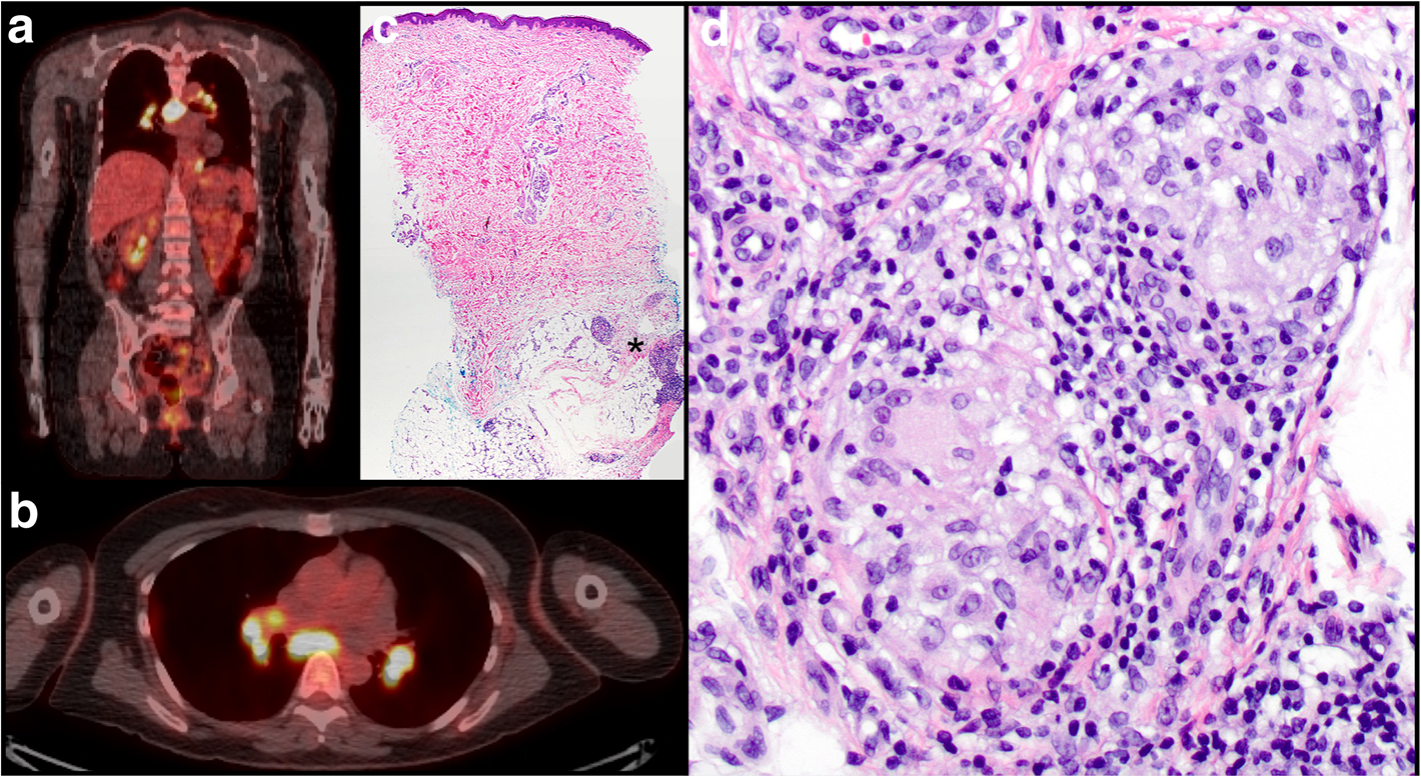
Patient 2. **a** Axial and **b** coronal views of PET/CT with FDG avid bilateral hilar and mediastinal lymph nodes. **c** Skin biopsy with epithelioid granulomata in the subcutaneous tissue (*) (H&E, original magnification × 20). **d** Collection of epithelioid histiocytes with surrounding lymphocytes inflammation forming non-caseating granulomata lacking caseating necrosis (H&E, original magnification × 400)

A 44-year-old woman with stage IIIB metastatic melanoma from the chest (Clark level IV, Breslow thickness of 0.37 mm, and mitotic rate of 1/mm^2^) with synchronous metastasis to 1 of 23 regional lymph nodes began ipilimumab therapy (3 mg/kg) in the adjuvant setting. The patient completed 4 cycles of ipilimumab with no adverse reactions. Imaging studies after completion of the 4 doses revealed new, nonspecific, metabolically active mediastinal and bilateral hilar LAD that appeared reactive, and clinical surveillance was continued. Three months after completing ipilimumab therapy, imaging studies revealed enlarged, FDG avid mediastinal and bilateral hilar lymph nodes (Fig. 2a-b) and small FDG avid foci in subcutaneous tissue of both lower extremities. Physical examination revealed 4 small palpable subcutaneous nodules of the bilateral peri-patellar skin, and histologic evaluation demonstrated a collection of epithelioid histiocytes in the subcutis, forming multiple granulomatous/sarcoid-like lesions associated with CPIs (Fig. 2c-d). Past medical history included hypothyroidism, hypertension, and partial hysterectomy. There was no history of an autoimmune disorder.

Eight months after completing ipilimumab, CT examination demonstrated interval reduction in the size of the mediastinal and hilar lymph nodes and no evidence of disease progression. On dermatologic examination, the peri-patellar skin lesions demonstrated complete resolution with no specific intervention.

### Patient 3

**Fig. 3:**
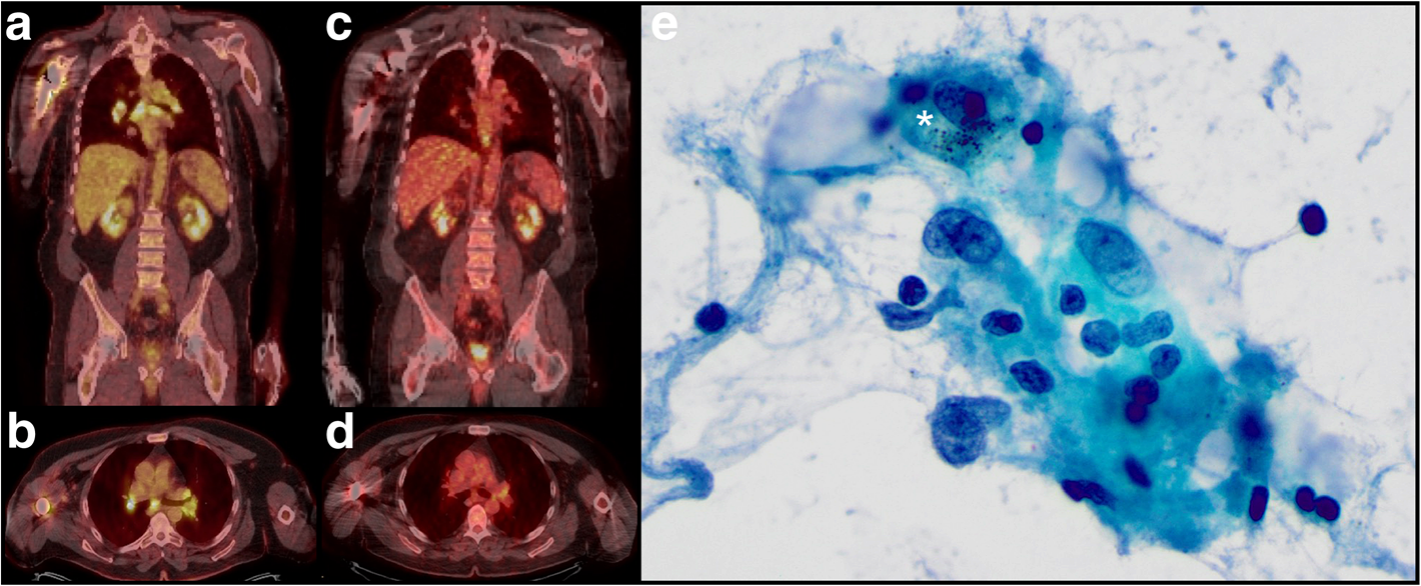
Patient 3. **a** Axial and **b** coronal views of PET/CT with FDG-avid hilar and mediastinal lymph nodes. **c** Axial and **d** coronal views of PET/CT with resolution of FDG-avid hilar and mediastinal lymph nodes after 18 months of immune checkpoint therapy. **e** Ultrasound guided endobronchial fine needle aspiration biopsy was negative for melanoma and revealed cluster of reactive epithelioid histiocytes, some with anthracotic pigmented macrophages (*), admixed with scattered lymphocytes (Papanicolaou stain, original magnification × 40)

A 68-year-old man with stage IV M1c metastatic *NRAS G13D*, *CDKN2A*, and *TP53* mutant, ulcerated lentigo maligna melanoma from the neck (Clark level IV, Breslow thickness 2.5 mm, mitotic rate of 16/mm^2^) developed disease recurrence in 1 of 17 ipsilateral neck lymph nodes without extracapsular extension 8 months after wide local excision and one negative sentinel lymph node biopsy. One and 3 months after disease recurrence, imaging studies revealed distant metastases to right proximal humerus and soft tissue (9.5 cm) and temporal lobe of brain, respectively. Pembrolizumab therapy (2 mg/kg) was initiated, and stereotactic radiosurgery with a gamma knife was performed on the solitary brain metastasis. After 6 months (9 doses) of pembrolizumab, the patient developed new hypermetabolic hilar and mediastinal lymph nodes (the largest measured 1.6 cm) on PET/CT (Fig. 3a-b) at the site of a non-tumor draining lymph node basin. These hilar and mediastinal lymph nodes were inconspicuous radiographically prior to pembrolizumab therapy, but developed only during the course of pembrolizumab therapy. Ultrasound-guided endobronchial fine needle aspiration (FNA) biopsy of targeted, hypermetabolic lymph nodes (subcarinal Station 7 and lower paratracheal 4R) were negative for melanoma (Fig. 3c). Instead, examination of FNA revealed these lymph nodes were reactive; containing clusters of epitheilioid histiocytes with pigmented macrophages. Restaging scans after 8 months of pembrolizumab therapy (13 doses) revealed resolution of PET avid mediastinal lymph nodes and no evidence of disease (Fig. 3d-e). Taken together, the clinical and radiographic presentation along with our prior experience was most compatible with granulomatous/sarcoid-like lesions associated with CPIs. Past medical history include non-melanocytic skin cancers. There was no history of an autoimmune disorder.

After nearly 2 years of pembrolizumab therapy (32 doses), there was no evidence of disease progression.

## Discussion and conclusions

Granulomatous/sarcoid-like lesions associated with CPIs may be clinically and radiographically concerning for disease recurrence and may significantly impact patient treatment [8, 9, 11]. Recognition and appropriate treatment of such toxicities resulting from immune checkpoint therapy are critical for optimal patient care. To date, a comprehensive review of the literature identified 26 patients (including 3 from this report) with a median age of 57 years (range: 26-79 years) who developed granulomatous/sarcoid-like lesions (*n* = 23, sarcoidosis-like reactions; *n* = 3, granulomatous panniculitis) associated with CPIs (ipilimumab = 14; nivolumab = 3; pembrolizumab = 5; anti-PD-L1 = 1; combined ipilimumab + nivolumab = 3) (Table 1) [7–11, 15–29]. There was slight female predominance (M:F ratio 11:13), and melanoma accounted for 77% of the primary tumors treated with immune checkpoint therapy. Interestingly, hypothyroidism was seen in a subset of patients with granulomatous/sarcoid-like lesions associated with CPIs. Sites most frequently involved by sarcoidosis include the lung, hilar and mediastinal lymph nodes, and/or skin (in 79% of cases). There were 4 patients with granulomatous/sarcoid-like lesions associated with CPIs were limited to the skin, including 3 patients with granulomatous panniculitis, of which, 2 had no clinical and radiographic features of LAD. The median duration of immune checkpoint therapy was 6 months (range: 0.75-20 months) in patients that developed granulomatous/sarcoid-like lesions associated with CPIs. Treatment for the granulomatous/sarcoid-like lesions associated with CPIs included withholding therapy in 38% of patients and/or administration of systemic steroids in 44% of patients. Either resolution or improvement of these immune-mediated reactions occurred in 96% of reported patients irrespective of how the toxicity was managed. Partial therapeutic response, stable disease, or complete remission of malignancy was observed in 71% of reported patients who developed granulomatous/sarcoid-like lesions associated with CPIs over a median follow-up of 11.5 months (range: 3-54 months) since initiation of treatment. Disease progression was observed in 29% of reported patients with granulomatous/sarcoid-like lesions associated with CPIs. Follow up was not available for 2 patients.

**Table 1:** Granulomatous/sarcoid-like lesions associated with checkpoint inhibitors

Case	Age (yrs)	Sex	Primary disease	Site of metastasis	Clinical presentation	Sites of granulomatous/sarcoid-like lesions	Immune checkpoint inhibitors (dose)	Onset of granulomatous/ sarcoid-like lesions after initiation of immune checkpoint inhibitors (months)	Histologic features	Treatment of granulomatous/sarcoid-like lesions (dose)	Outcome of granulomatous/ sarcoid-like lesions	Disease response to immune checkpoint inhibitors	Follow up since initiation of immune checkpoint inhibitor (months)
1 Anderson, 2014 [7]	44	M	Melanoma	Kidney, lungs, mediastinal and retroperitoneal LN, brain, bone, muscle, subcutis	Routine surveillance	Spleen	Ipilimumab (3 mg/kg, Q3WKS) Completed 4 cycles	20	Spleen biopsy: non-caseating epithelioid granulomata	None	Resolution of splenic lesion	Stable disease	33
2 Firwana, 2016 [15]	41	M	Melanoma Colorectal carcinoma	Axillary LN	Bilateral, occipital neck pain, axillary and cervical LAD	Bilateral cervical, axillary, hilar, mediastinal, iliac and inguinal LNs	Ipilimumab (NR)	NR	Not available	Ipilimumab withheld Opioids and NSAIDs	Resolution of LAD	NR	NR
3 Firwana, 2016 [15]	57	F	Melanoma	Chest wall	Flu-like symptoms, fatigue, skin with erythematous, painful nodules on lower extremity	Hilar, mediastinal LNs	Ipilimumab (NR)	NR	Hilar lymph node biopsy: poorly formed epithelioid granulomata with focal necrosis	Prednisone (1 mg/kg)	NR	NR	NR
4 Eckert, 2008 [16]	67	F	Melanoma	Axillary, supraclavicular, spinal, LNs, subcutis, liver	Low-grade dyspnea, skin lesion on face	Facial skin, mediastinal LNs	Ipilimumab (0.3, 3, or 10 mg/kg, Q3WKS + 10 mg/kg 3WKS)	7	Face and bronchial biopsy: non-caseating granulomata	Ipilimumab withheld	Partial resolution of LAD	Stable disease	11
5 Seve, 2009 [17]	62	F	Melanoma	Skin, liver	NR	NR	Ipilimumab (0.3, 3, or 10 mg/kg, Q3WKS)	7	Biopsy from skin and bronchus: consistent with sarcoidosis	Ipilimumab withheld	Resolution	Stable disease	54
6 Vogel, 2012 [18]	49	M	Melanoma	Bilateral inguinal, pulmonary, mediastinal LNs, bilateral legs	Routine surveillance	Mediastinal and bilateral hilar LAD	Ipilimumab (3 mg/kg, Q3WKS)	5	Endobronchial biopsy: non-caseating granulomatous inflammation	None	Resolution of LAD	Complete remission	~ 8
7 Wilgenhof, 2012 [19]	48	F	Melanoma	Bilateral lungs, mediastinum, axilla, retroperitoneal, breast	Dry cough, shortness of breath, fatigue	Skin of neck, axilla, mediastinum, and retroperitoneal LAD, lung, spleen	Ipilimumab (3 mg/kg, Q3WKS)	1	Transbronchial biopsy: non-necrotizing epithelioid granulomata	Completed 4 doses of Ipilimumab Methylprednisolone (48 mg)	Decrease in size of LAD and spleen	Progression of disease	9
8 Berthod, 2012 [20]	63	M	Melanoma	Lung, liver, mediastinal LNs	Dry cough and dyspnea	Lung, pleura, perihilar tissue	Ipilimumab (3 mg/kg, Q3WKS)	3.25	Bronchial and lung biopsies: well-formed granulomata with giant cells and occasional necrosis	Prednisone (1.5 mg/kg)	Resolution of lung infiltrates	Progression of disease	~ 6
9 Tissoot, 2013 [21]	57	M	Melanoma	Axilla	Subcutaneous nodules on arm	Skin, lung, bilateral hilar LNs	Ipilimumab (10 mg/kg, Q3WKS for 4 doses, followed by 10 mg/kg, Q12WKS)	9	Skin and mediastinal biopsy: non-caseating granulomata	Ipilimumab withheld	Skin and pulmonary lesions resolved, decreased size of mediastinal LNs	Remission	12
10 Reule,2013 [22]	55	M	Melanoma	Axilla	Grouped erythematous papules	Skin, lung, hilar and mediastinal LNs	Ipilimumab (10 mg/kg, Q3WKS)	1.5	Subcarinal lymph node biopsy: negative for malignancy Skin biopsy: sarcoidal granulomata	Prednisone	Rapid improvement	Progression of disease	NR
11 Murphy, 2014 [23]	37	M	Melanoma	Inguinal, pelvic LNs, vertebrae	Routine surveillance	Bilateral hilar and mediastinal LNs, brain	Ipilimumab (3 mg/kg, Q3WKS)	6.25	Transbronchial biopsy: non-caseating granulomata	Prednisolone (40 mg)	Resolution of LAD	Stable disease	12
12 Toumeh, 2016 [24]	26	F	Melanoma	Axilla, adrenal gland, subcutis	Intermittent abdominal pain	Skin, mediastinal LNs, lung, peritoneal surface of liver	Ipilimumab (3 mg/kg, Q3WKS)	1	Mediastinal LN biopsy: cohesive clusters of epithelioid histiocytes and multinucleated giant cells Skin biopsy: non-necrotizing granulomata associated with tattoo pigment	Prednisone (60 mg)	Near complete resolution of Mediastinal LAD Enlargement of peritoneal nodules, confirmed as melanoma	Progression of disease	4
13 Current report, Patient 2	33	F	Melanoma	Axilla	Routine surveillance/staging studies	Skin of bilateral lower extremities, mediastinal and hilar LNs	Ipilimumab (3 mg/kg, Q3WKS)	3	Skin biopsy: collection of epithelioid histiocytes	None	Improvement of LAD	Remission	8
14 van den Eertwegh, 2012 [25]	NR	NR	Prostate adenocarcinoma	NR	Surveillance studies	Lung	Ipilimumab (5 mg/kg, Q4WKS)	2	Lung biopsy: small, non-compact granulomata	Ipilimumab withheld Prednisone (NR)	Improvement	NR	NR
15 Suozzi, 2016 [8]	60	F	Lung adenocarcinoma	LNs, brain	Nausea, vomiting, aphasia, confusion	Skin with multiple pink firm papules and annular plaques	Ipilimumab (1 mg/kg, Q6WKS) + Nivolumab (1 mg/kg, Q2WKS)	7	Skin biopsy: dermal granulomatous inflammation	Clobetasol ointment (0.05%)	Some improvement	Progression of disease	10
16 Birnbaum, 2017 [9]	63	F	Lung adenocarcinoma	Pleura	N/A	Skin of neck, face, and periorbital, pruritic waxing and waning, erythematous papules and plaques	Nivolumab (3 mg/kg, Q2WKS)	4.5	Skin biopsy: nodular collection of epithelioid histiocytes with multinucleated giant cells	Methylprednisolone (24 mg) Hydroxychloroquine (200 mg)	Complete resolution	Stable disease	6
17 Montaudie, 2016 [26]	56	M	Melanoma	Axilla, hilar, mediastinal LNs, liver, lung,	Dry cough and dyspnea	Bronchi, lung, parotid glands, cervical LNs	Nivolumab (3 mg/kg, Q2WKS)	0.75	Bronchial biopsy: non-caseating epithelioid granulomata	Prednisone (75 mg)	Resolution of lung nodules	Progression of disease	3
18 Danlos, 2016 [27]	57	M	Melanoma	Skin, nasolabial fold	Surveillance studies	Cutaneous lip, subcutaneous tissue near prior scar, bilateral hilar, mediastinal LNs	Nivolumab (3 mg/kg, Q2WKS)	10	Biopsy of subcutaneous nodule: non-necrotizing epithelioid granulomata with giant cells and some birefringent material	None	Resolution of granulomata	Remission	12
19^a^ Cotlier, 2016 [28]	72	F	Hodgkin lymphoma	N/A	Enlarging asymptomatic nodules	Skin of upper extremities, axial skeleton, eye, lung, bilateral hilar and mediastinal LNs	Pembrolizumab (200 mg, Q3WKS)	6	Biopsy of skin nodule: focal dermal epithelioid granulomata	Pembrolizumab withheld Prednisone	Complete resolution of Skin nodules, LAD, and FDG-avid lesions	Remission	13
20 Firwana, 2016 [15]	37	F	Melanoma	Spleen, radius	Joint pain, cough, granulomatous lesions in the arm near surgical scar and bilateral skin nodules of lower extremity	Cervical, axillary, mediastinal, retroperitoneal LAD	Pembrolizumab (Not reported)	NR	Right lung wedge resection: sarcoidosis	Pembrolizumab withheld	Partial resolution of LAD	NR	NR
21 Current report, Patient 1	79	M	Melanoma	Hilar, mediastinal LNs, liver, adrenal	Subcutaneous nodules	Skin, mediastinal, hilar, peritracheal, retroperitoneal	Pembrolizumab (2 mg/kg, Q3WKS)	20	Biopsy of skin nodule: non-caseating epithelioid granulomata	Pembrolizumab withheld Intralesional triamcinolone	Persistence of skin nodules, stable LAD	Remission	25
22 Current report, Patient 3	68	M	Melanoma	LNs, skin, humerus, brain	Surveillance studies	Mediastinal, paratracheal LAD	Pembrolizumab (2 mg/kg, Q3WKS)	6	Biopsy of paratracheal lymph nodes: “negative for melanoma”	None	Resolution of LAD	Remission	24
23 Brahmer, 2012 [29]	NR	NR	Melanoma	Lymph node	NR	NR	Anti-PD-L1 (10 mg/kg, Q2WKS)	NR	NR	NR	NR	NR	NR
24 Burillo-Martinez, 2017 [10]	60	F	Melanoma	Peritoneal	Plaques, nodules	Skin of upper and lower extremity, bilateral hilar and mediastinal LAD	Pembrolizumab (2 mg/kg, Q3WKS)	1.2	Biopsy of skin nodule: lobular granulomatous panniculitis	Pembrolizumab withheld Prednisone	Complete resolution	Complete remission	6
25 Tetzlaff, 2017 [11]	57	F	Ovarian cancer	Liver, peritoneum	Tender subcutaneous nodules	Skin of upper and lower extremities	Nivolumab (3 mg/kg, Q2WKS) + Ipilimumab (1 mg/kg, Q6WKS)	10	Biopsy of skin nodule: septal and lobular panniculitis with giant cells and septal fibrosis	None	Complete resolution	No evidence of disease	16
26 Tetzlaff, 2017 [11]	39	F	Melanoma	Axillary LNs	Tender subcutaneous nodules	Skin of lower extremities, buttock	Neoadjuvant setting: Nivolumab, 3 doses (1 mg/kg, Q3WKS) + Ipilimumab, 3 doses (3 mg/kg, Q3WKS) Adjuvant setting: Nivolumab (3 mg/kg, Q2WKS)	7	Biopsy of skin nodule: septal and lobular panniculitis with giant cells and septal fibrosis	All melanoma therapy withheld Systemic steroids Hydroxychloroquine	Nodules dissipated	No evidence of disease	12
Total	Median (range) 57 (26-79)	M:F^b^ 11:13	Melanoma = 20 Other =6^c^			Skin, lung, and hilar/mediastinal LNs = 10 Lung, hilar/mediastinal LNs = 9 Skin only = 4 Other = 6^d^	Ipilimumab = 14 Nivolumab = 3 Pembrolizumab = 5 Anti-PD-L1 = 1 Combined ipilimumab + nivolumab = 3	Median (range) 6 (0.75-20)		Immune checkpoint inhibitor withheld = 10 Systemic steroids = 12 Localized = 2	Resolution =14 Improvement = 9 Stable = 1 NR =2	Stable = 5 Remission = 10 Progression =6 NR =5	Median (range) 11.5 (3-54)

*Abbreviations: M* male, *F* female, *NR* not reported, *LN* lymph nodes, *LAD* lymphadenopathy, *Q* every, *WKS* weeks

^a^History of sarcoidosis before immune checkpoint therapy

^b^Two patients’ information was not reported

^c^One patient had both melanoma and colorectal carcinoma

^d^Some patients had multiple sites of sarcoidosis. Other sites included spleen, parotid gland, axial skeleton, eye, cervical and retroperitoneal lymph nodes

Patients with prolonged chronic inflammation and immune dysfunction have increased risk of developing, lymphomas, melanomas and other solid tumors as well as sarcoidosis [14, 30]. Furthermore, cancer patients with sarcoidosis have higher incidence of melanoma, hematolymphoid, gastrointestinal, and other solid tumors [14]. However, activation of T-cell response with CPI therapy has improved survival in a subset of patients with these types of malignancies. In addition, it appears a subset of patients who develop granulomatous/sarcoid-like lesions during CPI therapy exhibit favorable therapeutic response. Therefore, the balance of T-cell activation and type of T helper (Th) response appears complex.

Cancer therapy associated granulomatous/sarcoid-like lesions is not unique to immune checkpoint therapy and has been described in a variety of patients with hematologic and solid tumor malignancies, including melanoma that were treated with alpha interferon, methotrexate, cisplatin, interleukin 2, and vemurafenib [14, 31]. Historically, augmentation of the patient’s immune response with intralesional bacilli Calmette-Guerin (BCG) for melanoma therapy appeared promising with clinical benefits in some patients [32]. BCG therapy associated activation of melanoma specific and nonspecific immune response induced the development of granulomata. Serial biopsies taken of lesions at early (24 h), middle (2-3 weeks), and late (> 3 weeks to ≤2 months) time points after BCG administration, exhibited distinct histomorphology [32]. Early lesions had inflammatory response with polymorphonuclear leukocytes, lymphocytes, and mononuclear cells towards the periphery with some disruption of melanoma cells and pigment release. Examination of lesions taken during the middle course of BCG therapy, showed early granuloma formation with macrophages, lymphocytes, and giant cells, and late lesions exhibited organized, well form non-caseating granulomata [32]. Parallel with BCG therapy, CPIs attempt to restore patient’s immune function by blocking inhibitory signals with anti-CTLA-4 or anti-PD-1/PD-L1. Similar granulomatous/sarcoid-like reactions are being now being recognized with the current class of CPIs. Interestingly, in our review of granulomatous/sarcoid-like lesions associated with checkpoint inhibitors, this finding occurred in more than three quarters of the patients under therapy for metastatic melanoma. Melanomas are highly immunogenic and the neoantigen landscape in melanoma cells poses a significant impact on anti-tumor activity of T-cells and response to immune checkpoint therapy [33, 34]. Activation of cytotoxic T-cells and enhanced killing of melanoma cells after treatment with CPIs may possibly expose additional neoantigens presented by antigen presenting cells that promote Th1 response and cytokine milieu favorable to develop granulomatous/sarcoid-like lesions during CPI therapy.

Various types of dermatologic toxicities to immune checkpoint therapy can be grouped as inflammatory processes, reactions that target keratinocytes, and affect melanocytes. Immune-mediated reactions that target melanocytes may manifest as vitiligo and/or regression of nevi have been associated with favorable therapeutic response to immune checkpoint therapy [5, 35]. The development of granulomatous/sarcoid-like lesions associated with CPIs appear to be another irAE associated with a favorable therapeutic response in a subset of patients treated with immune checkpoint antibodies (Table 1).

The immunopathogenesis of sarcoidal granulomata involve the interaction of monocyte-derived histiocytes and CD4+ Th1 cells [12]. An array of cytokines and chemokines is involved in the development of sarcoidosis, although Tumor Necrosis Factor-α (TNF-α) is thought to be central to the formation and maintenance of these “sarcoidal” granulomata [12]. Thus, monoclonal antibodies to TNF-α (i.e., infliximab and adalimumab) have been successful in the treatment of sarcoidosis [36, 37]. Intriguingly, some patients who received anti-TNF-α therapy for the management of their inflammatory bowel disease, psoriasis, arthritis, or sarcoidosis developed paradoxical sarcoidosis-like reactions to therapy [29, 38–41]. Investigators have suggested that anti-TNF-α therapy may impair the regulatory role of TNF-α on autoreactive T cells, resulting in a cytokine imbalance and promoting Th1 immune response [42].

There are competing paradigms in the immunopathogenesis of sarcoidosis that either involve a hypoactive or hyperactive immune response [9]. Patients who develop sarcoidosis unrelated to immune checkpoint therapy are thought to have a hypoactive immune response with decreased T-cell proliferation and PD-1 up-regulation [43]. In contrast, those who develop sarcoidosis related to immune checkpoint blockade would support a hyperactive immune response with increased T-cell proliferation and inhibition of PD-1 signaling [9]. The extent of the disruption of mediators in homeostasis and cytokine imbalance may promote the development of granulomatous/sarcoid-like lesions associated with CPIs that exhibits granulomatous panniculitis features with multinucleated giant cells to “sarcoidal” granulomata [11].

There are variety of terminologies utilized in the literature (Table 1) that describe manifestation of granulomatous/sarcoid-like lesions associated with CPIs and include: “sarcoidosis”, “cutaneous sarcoidosis”, “sarcoidosis-like syndrome”, “sarcoid-like granulomatosis” and among others. To avoid confusion with disease of systemic sarcoidosis, unrelated to CPIs, we suggest the term “granulomatous/sarcoid-like lesions associated with CPIs” involving the organ type (e.g. skin, lungs, etc). This term will encompass all granulomatous reactions (e.g. granulomatous panninculitis) that occur during CPI therapy as well as lesions with granulomas that morphologically appear “sarcoidal”. This terminology will also incorporate previously described reports in the literature of “sarcoid” granulomas from CPI therapy.

Sarcoidosis is a systemic disease and remains a diagnosis of exclusion. A presumptive diagnosis of sarcoidosis may be made on the basis of clinical and radiographic features [44]. Bilateral hilar lymphadenopathy is the most common finding and the patients may be asymptomatic or may present with nonspecific skin lesions of erythema nodosum–like panniculitis [14]. Alternatively, the patient may present with a triad of uveitis, parotiditis, and fever or with abnormal gallium imaging with increased uptake in the parotid and lacrimal gland and right paratracheal and bilateral hilar uptake [44].

The development of granulomatous/sarcoid-like lesions associated with CPIs is an immune-mediated toxicity that may clinically and radiographically mimic disease recurrence and/or progression. Recognition of this type of toxicity is important for appropriate management and possible measurement of therapeutic response in a subset of patients that manifest this type of immune-mediated adverse event.

## Abbreviations

CPIs: Checkpoint inhibitors
CT: Computed tomography
CTLA-4: cytotoxic T-lymphocyte–associated protein-4
FDG: Fludeoxyglucose
irAE: Immune-related adverse events
LAD: Lymphadenopathy
PD-1: Programmed cell death protein 1
PET/CT: Positron emission tomography/computed tomography
Th: T helper
TNF-α: Tumor Necrosis Factor-α
